# The potential for oral combination chemotherapy of 5′-deoxy-5-fluorouridine, a 5-FU prodrug, and cyclophosphamide for metastatic breast cancer

**DOI:** 10.1038/sj.bjc.6601350

**Published:** 2003-10-28

**Authors:** M Yoshimoto, K Tada, N Tokudome, G Kutomi, M Tanabe, T Goto, S Nishimura, M Makita, F Kasumi

**Affiliations:** 1 Breast Oncology Group, Cancer Institute Hospital, Kami-Ikebukuro 1-37-1, Toshima-ku, Tokyo 170-8455, Japan

**Keywords:** breast cancer, chemotherapy, oral, doxifluridine, capecitabine

## Abstract

Preclinical studies have demonstrated the synergistic anti-tumour activity of combination therapy with the oral cytostatics, 5′-deoxy-5-fluorouridine (5′-DFUR) and cyclophosphamide (CPA), in human breast cancer xenograft models. This study was performed to evaluate the efficacy and safety of this oral combination chemotherapy in the treatment of metastatic breast cancer. In all, 101 patients with metastatic breast cancer were enrolled in the study, and the data for 94 eligible patients of these were evaluated. The patients received twice daily oral combinations of 5′-DFUR (1200 mg/body/day) and CPA (100 mg/body/day) for 2 weeks, followed by a 1-week rest period. After a median of 19 treatment cycles (range 1–66 cycles), 16 patients (17.0%) had a complete response, and 40 patients (42.6%) had partial responses. The response rate was 59.6% (95% CI, 49.0–69.6%). The median time to progression and overall survival times were 11.7 and 40.3 months, respectively. The toxicity was mild and tolerable, and the related grade 3/4 clinical adverse effects consisted of haematological toxicity in 21 patients (22%) and nonhaematological toxicity in five patients (5%). These results suggest that the oral combination chemotherapy of 5′-DFUR and CPA has low toxicity and is a novel, very convenient and effective treatment for metastatic breast cancer.

Metastatic breast cancer is generally considered to be incurable and conventional cytotoxic chemotherapy is used mainly for palliation (Henderson *et al*, 1989). Standard chemotherapeutic regimens for metastatic breast cancer utilise drugs such as anthracyclines that are widely used and taxanes, (Greenberg *et al*, 1996; Burris, 1999; Nabholtz *et al*, 1999). Although standard combination chemotherapy with anthracyclines and taxanes results in significant response rates in first-line usage, the duration of this response is short lived and the percentage of patients in whom this treatment results in a cure is extremely low, even when a complete response is obtained (Greenberg *et al*, 1996; Nabholtz *et al*, 1999). When the standard regimens fail, there are a few other effective chemotherapeutic regimens. The advent of novel therapeutic regimens with reduced toxicity is therefore highly desirable.

5′-deoxy-5-fluorouridine (5′-DFUR, doxifluridine), an intermediate metabolite of capecitabine (Xeloda®), is an orally administered prodrug of fluorouracil (Cook *et al*, 1979; Ishitsuka *et al*, 1980; Miwa *et al*, 1998; Ishitsuka, 2000), which exerts its anticancer activity after metabolic conversion to 5-flourouracil (5-FU) by pyrimidine nucleoside phosphorylase (PyNPase, thymidine phosphorylase: TP in humans) (Kono *et al*, 1983), a strong angiogenic factor identical to platelet-derived endothelial cell growth factor (PD-ECGF) (Furukawa *et al*, 1992). There are higher levels of this enzyme in the neoplastic tissue than in the normal tissue (Nishida *et al*, 1996; Takebayashi *et al*, 1996), and 5′-DFUR therefore exhibits selective activity against various neoplasms while having relatively low systemic toxicity (Niitani *et al*, 1985; Uehara *et al*, 1985; Ishikawa *et al*, 1995). The response rate with 5′-DFUR monotherapy for the treatment of metastatic breast cancer has been reported to be 35.9% (Niitani *et al*, 1985).

Sawada and Endo *et al* discovered that particular cytostatics, such as taxanes, mitomycin C (MMC) and cyclophosphamide (CPA), up-regulated TP expression preferentially in the tumour tissue when therapeutic dose levels of these drugs were administered, even after oral CPA administration, whereas TP was not affected in normal tissues including the small intestine and liver. Also, they demonstrated that combinations of 5′-DFUR/capecitabine and these cytostatics exhibited excellent synergistic activity against various human cancers in xenograft models, without significant potentiation of toxicity (Sawada *et al*, 1998; Endo *et al*, 1999). Such synergistic activities were not recognised in combination with 5-FU nor UFT (a mixture of tegaful and uracil) and these cytostatics (Endo *et al*, 1999). Of these cytostatics, we were particularly interested in the combination of 5′-DFUR and CPA as both of these drugs can be administered orally (Yoshimoto *et al*, 1998).

A combination chemotherapy of 5′-DFUR and CPA has been reported to be more effective in preventing tumor recurrence than 5′-DFUR alone in an adjuvant setting (Tominaga *et al*, 2003), but the definitive evidence of the efficacy of this combination for metastatic breast cancer has yet to be published. Based on these previously reported preclinical and clinical results, we devised a study wherein a combination of the two drugs was orally administered to patients with metastatic breast cancer.

## PATIENTS AND METHODS

### Inclusion and exclusion criteria

Patients with the following criteria were included: patients with bidimensionally measurable or assessable metastatic breast cancer including inoperable stage IV breast cancer that had been histologically, cytologically, or radiologically confirmed; patients who had undergone treatment with a maximum of one previous chemotherapy regimen for metastatic disease; patients with progressive osseous metastases were eligible, but patients with only osseous metastasis who had received radiotherapy were excluded from the study. There were no age restrictions for this study.

Patients with the following criteria were excluded: World Health Organization (WHO) performance status greater than three, except for cases of osseous metastasis; brain or leptomeningeal metastases; serious cardiac, liver, kidney, or central nervous system disease that precluded the treatment; a white blood cell count less than 3000 cells *μ*l^−1^, haemoglobin less than 9.0 g dl^−1^ or a platelet count less than 100 000 cells *μ*l^−1^.

Patients were required to have passed a minimum of 4 weeks after administration of their most recent chemotherapy, hormone therapy, or radiation therapy, and to have recovered from all treatment-related toxicity. Where possible, a histological or cytological confirmation of diagnosis was performed.

### Dose setting

The optimal maximum dosage for 5′-DFUR monotherapy was approved as 1200 mg/body/day by Japan's Ministry of Health and Welfare at 1987, but sufficient rest periods (1 or 2 weeks) at intervals of every 2 weeks of administration were also recommended to prevent diarrhoea, a possible serious side effect of 5′-DFUR. Based on the initial dose setting, Ota *et al* found that a combination therapy of 5′-DFUR at doses of 1200 mg/body/day with CPA at 100 mg/body/day for 2 weeks with a 2-weeks rest period was safe and tolerable in a preliminary early Phase II trial (Ohta *et al*, 1992). Tominaga *et al* developed a Phase III trial comparing 5′-DFUR plus CPA with 5′-DFUR alone with the same dose setting with or without tamoxifen in an adjuvant setting, and revealed that the regimen was feasible and safe enough, causing only 4.1% of grade ≥3 side effects (Tominaga *et al*, 2003). Experimental xenograft models, however, showed that 2-weeks rest period was too long, whereas a 1-week rest period was adequate, because the elevation of TP levels in the tumour tissue by CPA was maintained over 1 week or less (Endo *et al*, 1999). In this study, then, we adopted a dose setting as a daily oral combinations of 5′-DFUR (1200 mg/body) and CPA (100 mg/body) for 2 weeks, followed by a 1-week rest period.

### Treatment plan

This open-label Phase II trial, approved by the hospital's institutional review board (IRB), was performed in a single institute and was designed to evaluate the safety and efficacy of orally administered combination chemotherapy of 5′-DFUR and CPA.

All patients were fully informed of the potential benefits and potential side effects of the proposed treatment, and gave written informed consent to participate in the study prior to the start of the study. If side effects were tolerable, the treatment was continued until progression of the disease was recognised. No other cytostatic agents or bisphosphonates were used during the course of the treatment.

Patients were instructed to immediately stop taking the medication if any kind of unusual diarrhoea or appetite loss occurred. In such cases, treatment was resumed with a decreased 5′-DFUR dosage of 800 or 600 mg/body/day, without changing the CPA dosage, after a sufficiently long rest period, usually 2 weeks. If the resumed treatment with the decreased dose of 5′-DFUR induced the same side effects, the treatment was discontinued. For patients in whom the absolute white blood cell count dropped to 2000 *μ*l^−1^ or less, the rest period was extended by 1 week to a total of 2 weeks.

Patients received treatment until disease progression or a maximum of 3 years in the case of continuing complete response to avoid the deterioration of bone marrow function and/or the possibility of other serious side effects. Where possible, hormonal maintenance treatments were allowed after this period.

Proper treatments after the progression of disease was not determined in the study, but anthracycline-containing regimens and/or taxanes were the usual treatment options administered after progression of the disease if these drugs had not been previously used.

### Examination

Prior to the start of treatment, patients had their complete medical history documented and had a physical examination, including the measurement of tumour lesions, complete blood cell count, serum chemistry, serum tumour markers (CEA and CA15-3), chest X-ray, computed tomography or ultrasound examination of the liver, ECG, and bone scintigraphy if clinically indicated. During the study, on the first day of every treatment cycle, physical examinations were performed and vital signs, complete blood cell counts, serum chemistries and serum tumour markers were determined. Tumour assessments using X-ray, CT scan, or ultrasonography were performed every 6 weeks during the first 6 months and every 12 weeks after that period if the tumour was considered to be stable. If at any time there was a significant increase in the levels of serum tumour markers, a precise assessment of the state of the tumour was performed. Tumour assessments were also performed at the time of withdrawal from the study.

### Evaluation of response

Patients were considered assessable for response if they were enrolled with accuracy, underwent the treatment under the protocol, and had the first cycle of the treatment. The primary efficacy end point was overall objective tumour response (complete response (CR) and partial response (PR)), assessed using the International Union Against Cancer (UICC) criteria (Monfardini *et al*, 1987). The response for each patient was verified by independent blinded peer review by radiologists. This peer review assigned an assessment of overall tumour response: CR, PR and stabilisation of the disease (no change, NC), progressive disease (PD), or not assessable. Complete response, PR and NC had to be confirmed on two occasions at least 4 weeks apart (generally, at the next 6-week assessment).

A CR was defined as the total disappearance of clinically and radiologically detectable disease for patients with measurable disease, but was not defined for patients with unmeasurable lesions such as osseous lesions. A PR was defined as at least a 50% decrease in the sum of the product of the maximum perpendicular diameters for all existing lesions, with no new lesions appearing. Osteolytic bone lesions were regarded as assessable, and a PR in osseous lesions required a partial decrease in the size of lytic lesions and definite osteoblastic changes in osteolytic lesions to be observed on an X-ray film. An NC was defined by a reduction of less than 50% or an increase of less than 25% in the sum of the product of the maximum perpendicular diameters of all existing lesions, with no new lesions appearing. Among NCs, patients for whom the cancer remained stable for over 6 months were defined as long NC. A PD was defined if any new lesion appeared or if any existing lesion increased by 25% or more. A PD for bone metastasis was defined when increased in size of extent lesions or the appearance of new lesions. Response rates were retrospectively evaluated with respect to menopausal status, receptor status of the primary tumour, disease-free intervals, main sites of metastasis for evaluation, prior exposure to cytostatics, and the number of prior chemotherapy regimens for metastatic breast cancer.

The other end points included time to progression (TTP) of disease, and overall survival (OS) for the whole enrolled patients. The duration was calculated using the standard WHO criteria. Time to progression was determined as the interval between the day on which treatment was initiated and the earliest day that the progression was recognised, or the day of death from any cause. The time to response was determined for patients who obtained CR or PR as the interval between the day on which treatment was initiated and the earliest day that a response was recognised. The duration of the response was determined as the interval between the day that the response was recognised and the earliest day that disease progression was recognised, or the day of death from any cause.

### Evaluation of toxicity

Toxicity was evaluated for eligible patients according to the May 1998 revision of the National Cancer Institute (NCI) common toxicity criteria grading system.

### Statistical analysis

Time to progression and OS were estimated by the Kaplan–Meier product–limit method. The overall objective tumour response rates (CRs and PRs/whole) and clinical benefit rates (CRs, PRs and long NCs/whole) were analysed by logistic regression, presenting odds ratio with 95% confidence intervals (CI). *P*-values in *χ*^2^ tests of less than 0.05 were considered statistically significant.

## RESULTS

### Patient and tumour characteristics

From June 1995 to December 2000, 101 patients with metastatic breast cancer were enrolled and treated; of these 94 patients were deemed to be eligible for analysis. The remaining seven patients were judged as not eligible and were not included in the analysis for the following reasons: three patients with osseous metastasis were judged to have unevaluable lesions, four patients received other concomitant therapy (hormonal therapy, or radiation therapy, or administration of bisphosphonate agents for bone metastases).

The patient characteristics for the eligible patients are listed in Table 1

**Table 1:** Patient characteristics

**Characteristics**	**No.**	**%**
Age (years)		
≦40	7	7
41–50	22	23
51–60	36	38
61–70	21	22
≥71	8	9
Mean (range)	55.5 (31–77)	
		
*Menopausal status*		
Pre-	20	21
Post-	74	79
		
*Receptor status*		
ER-positive	44	47
-negative	24	26
-unknown	26	28
PgR-positive	37	39
-negative	29	31
-unknown	28	30
		
*Performance status*		
0	75	80
1	12	13
2	4	4
≧3	3	3
		
*Disease-free interval*		
≦2	36	38
2–5 years	36	38
>5 years	22	23
		
*No. of metastatic organs*		
1	67	71
2	24	26
≥3	3	3
		
*Main sites of metastasis*		
Skin/chest wall	16	17
Lymph nodes	16	17
Bone	24	26
Lung	21	22
Liver	16	17
Pleura (massive)	1	1
		
*Prior exposure to cytostatics*		
Hormonal only	14	15
Chemo only	34	36
Chemo+hormonal	34	36
None	12	13
		
*No. of prior chemotherapy regimens for MBC*		
0	68	72
1	26	28

ER=oestrogen receptor; PgR=progesterone receptor; MBC=metastatic breast cancer.

. The main sites of metastasis for evaluation were the local skin and/or the chest wall in 16 patients (including three patients with lung metastasis and two patients with bone metastasis), lymph node in 16 patients (including one patient with lung metastasis, and one patient with bone metastasis), bone in 24 patients, lung in 21 patients, liver in 16 patients and massive pleural metastasis in one patient. In all, 68 patients (72%) had one or more visceral sites of metastasis, and 23 patients (24%) had two or more sites of metastasis. A total of 62 patients (66%) had prior exposure to both 5-FU and CPA in the form of CMF (CPA, methotrexate and 5-FU) or CAF (CPA, doxorubicin and 5-FU), and 12 patients (13%) had prior exposure to anthracyclines.

### Efficacy

After a median of 19 treatment cycles (range 1–66 cycles), there were 16 (17.0%) CRs, 40 (42.6%) PRs, and 20 (21.3%) NCs (Table 2

**Table 2:** Tumour responses with regard to clinical status

		**Response**							
**Clinical status**	**No. of patients**	**CR**	**PR**	**NC (long NC)**	**PD**	**Response rate (%) (CR+PR/whole)**	**95% Confidence interval**		**Significance**
Whole eligible patients	94	16	40	20 (13)	18	59.6	49.0–69.6		—
									
*Menopausal status*									
Pre-	20	6	7	2 (0)	5	65.0	40.1–84.6		NS
Post-	74	10	33	18 (13)	13	58.1	46.1–69.5		
									
*Receptor status*									
ER-positive	44	9	23	7 (4)	5	72.7	54.8–83.2	}	P<0.05
-negative	24	2	8	6 (5)	8	41.7	22.1–63.4		
-unknown	26	5	9	7 (4)	5	53.8	33.4–73.4		
PgR-positive	37	9	21	5 (3)	2	81.0	64.8–92.0	}	P<0.05
-negative	30	2	9	8 (6)	11	37.9	19.9–56.1		
-unknown	27	5	10	7 (4)	5	53.6	35.3–74.5		
									
*Disease-free interval*									
≤2 years	36	5	13	8 (6)	10	50.0	32.9–67.1		NS
2–5 years	36	5	18	7 (3)	6	63.9	46.2–79.2		
>5 years	22	6	9	5 (4)	2	68.2	45.1–86.1		
									
*Main sites of metastasis*									
Skin/chest wall	16	4	3	7 (5)	2	43.8	19.8–70.1		NS
Lymph nodes	16	4	6	2 (1)	4	62.5	35.4–84.8		
Bone	24	0	16	5 (3)	3	66.7	44.7–84.4		
Lung	21	4	7	4 (3)	6	52.4	29.8–74.3		
Liver	16	4	7	2 (1)	3	68.8	41.3–89.0		
Pleura (massive)	1	0	1	0	0	100			
									
*Prior exposure to cytostatics*									
Hormonal only	14	0	8	4 (4)	2	57.1	28.9–82.3		NS
Chemotherapy only	34	7	12	7 (4)	8	55.9	37.9–72.8		
Chemo+hormonal	34	8	13	8 (4)	5	61.8	43.6–77.8		
None	12	1	7	1 (1)	3	66.7	34.9–90.1		
									
*No. of prior chemotherapy regimens for MBC*									
0	68	14	30	14 (9)	10	64.7	52.2–75.9		NS
1	26	2	10	6 (4)	8	46.2	26.6–66.6		

CR=complete response; PR=partial response; NC=no change; PD=progressive disease; ER=oestrogen receptor; PgR=progesterone receptor; MBC=metastatic breast cancer.

). Among NCs, the disease status of 13 patients remained stable for longer than 6 months (long NC). The overall response rate (CR+PR/whole) was 59.6% (95% CI, 49.0–69.6%). The response rate for first-line chemotherapy for metastatic disease in 68 patients was 64.7% (95% CI, 52.2–75.9%), including 14 CRs (20.6%). The response rate for 26 patients as a second-line chemotherapy was 46.2%. The overall clinical benefit rate (CR+PR+long NC/whole) was 73.4% (95% CI, 63.3–82.0 %).

**Figure 1:**
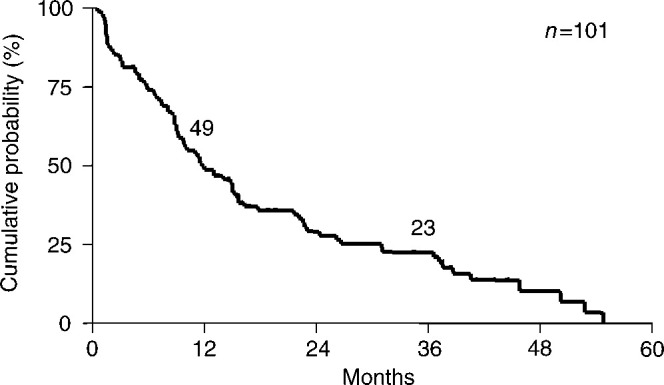
Time to progression (TTP) curves for whole patients.

**Figure 2:**
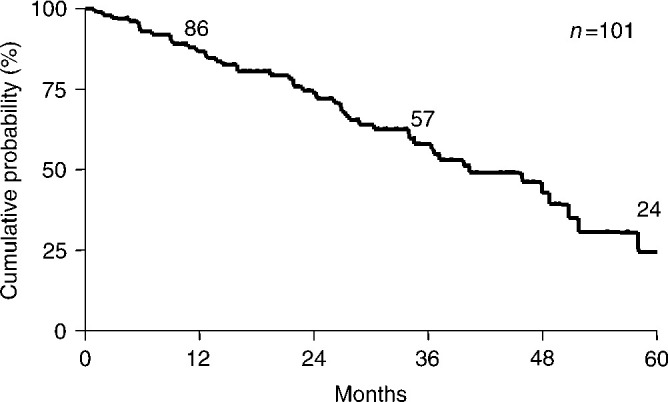
Overall survival curve for whole patients after the beginning of the treatment.

Among the enrolled whole 101 patients, 82 patients had progressive disease at the end of December 2001. Of the remaining 19 patients who had not progressed, nine remained as CRs and six remained as PRs. In all, 48 patients had died and 53 patients were alive. All, but one patient, who had CRs are alive after a median follow-up time of 36.8 months. The median (50%) TTP was 11.7 months, and 1- and 3-year TTPs were 49 and 23%, respectively (Figure 1

). The median OS was 40.3 months, and 1-, 3- and 5-year overall survival rates were 86, 57 and 24%, respectively (Figure 2

). The median TTP and OS for patients, in whom the treatment was used as first-line chemotherapy, were 12.7 and 45.6 months, respectively. For responders, the median time to response was 4.0±0.4 months (range 0.7–15.0 months). The median response duration was 17.4±1.8 months (range 1–52+ months).

Tumour responses with regard to clinical status are shown in Table 2. In all, 11 (68.8%) of 16 patients with liver metastasis responded to the treatment, including four patients who had a CR for 9–32 months. For the 62 patients with prior exposure to 5-FU and CPA in the form of CMF or CAF, a 59.7% response rate was obtained. The response rates for patients with oestrogen and/or progesterone receptor positive were as high as 72.7 and 81.0%, respectively, although all the hormone-receptor positive patients were hormone refractory. Also, there were significant differences in the response rate with regard to the receptor status of the primary tumour. In total, 12 patients with prior exposure to anthracyclines had a 50% response rate.

### Toxicity

The oral combination chemotherapy was well tolerated by most patients. The main toxic side effects are shown in Table 3

**Table 3:** Toxicities

	**CTC Grade (NCI, May 1998)**				
**Variable**	**1**	**2**	**3**	**4**	**% (Grade 3+4)**
*Haematological toxicity*					
Leucocytopenia	27	41	19	0	20.2
Granulocytopenia	21	39	16	0	17.0
Anaemia	14	18	3	0	3.2
Thrombocytopenia	25	5	0	0	0.0
					
*Digestive toxicity*					
Diarrhoea	6	2	4	0	4.3
Appetite loss	14	6	4	0	4.3
Nausea	12	3	3	—	3.2
					
Pulmonary toxicity	0	0	0	1	1.1
					
Liver and renal toxicity	21	0	0	0	0.0
					
Alopecia	0	0	—	—	—

.

Haematological toxicity was observed, but was generally mild and progressed slowly; only 21 (22%) patients had developed grade 3 toxicity. Grade 4 haematological toxicity was not observed in any patient. Diarrhoea and/or appetite loss were the most serious side effects, however, these side effects were observed in four patients, of whom two were successfully managed with a reduction of the 5′-DFUR dosage to 800 or 600 mg/body/day, without changing the CPA dosage after a sufficiently long period of rest. Grade 4 pulmonary toxicity occurred in one patient who developed interstitial pneumonia and died. This patient had previously undergone long-term treatment with CPA in the form of CMF for 2.5 years. Hand-foot syndrome, a common toxic side effect of capecitabine, was not observed. Significant liver or renal function toxicity was not observed, and cardiac insufficiency or alopecia were not observed.

Granulocyte colony-stimulating factor (G-CSF) nor special antiemetic drugs (such as antiserotonin agents) were not necessary to use during the course of treatment.

## DISCUSSION

Our results show that the oral combination chemotherapy of 5′-DFUR and CPA was not only convenient, but also effective in the treatment of metastatic breast cancer with mild side effects. The overall response rate of 59.6% (64.7% for first-line usage), the CR rate of 17.0% and the median TTP of 11.7 months match the values obtained by standard polychemotherapy regimens with anthracyclines (Rahman *et al*, 1999), previously reported as 65.0%, 16.6% and 11.5 months, respectively. The median survival time of 40.3 months is, however, significantly longer than the 21.3 months they reported.

It is thought that these results are partly due to the presence of some synergistic activity in the combination therapy of 5′-DFUR and CPA, because a high response rate of 59.7% was obtained in patients who had prior exposure to both 5-FU and CPA in the form of CMF or CAF. It may also be due to the fact that both 5′-DFUR and CPA are less toxic and less immunosuppressive than standard polychemotherapy regimens (Ohta *et al*, 1980; Tanaka *et al*, 1990). In addition, both the drugs can be administered for long periods of time (longer than 2 or 3 years) with mild side effects, as both drugs have different dose-limiting toxicities, digestive toxicity with 5′-DFUR and haematological toxicity with CPA. The two drugs also exhibit different kinds of anticancer activities, with 5′-DFUR having activity against CPA-resistant cancer cells (Endo *et al*, 1997). This combination therapy may have a non-cross-resistant anti-cancer activity with anthracyclines (Table 2) and/or with taxanes (data not shown). A combination of these factors may help to explain the high response rate and significantly longer median survival times observed in this study.

We have demonstrated that this treatment is effective, and that oral therapy offers many advantages over the standard infusion chemotherapy. The convenience of oral chemotherapy increases the treatment options for many patients with metastatic breast cancer. The reduction in side effects and the lack of severe nausea and vomiting, general fatigue, cardiac insufficiency and alopecia, which are usually caused by the standard infusion chemotherapy, helps patients to maintain their quality of life. An additional advantage of this therapy is that it should reduce the costs associated with the treatment of metastatic breast cancer, as it does not require hospital admission, rescue treatments such as G-CSF, or antiserotonin agents. In addition, mild and slow decreases in the bone marrow function allow for relatively longer intervals between monitoring examinations.

Experimental researches with regard to TP upregulation, preferentially in tumour tissue by taxanes, MMC and CPA (Sawada *et al*, 1998; Endo *et al*, 1999), propose the possible potentiality of the combination therapy of these cytostatics and 5′-DFUR or capecitabine. 5′-DFUR and CPA therapy by Yoshimoto *et al* (1998), and capecitabine and taxotere (TXT) therapy by O'Shaughnessy *et al* (2002), for breast cancer showed successful clinical examples. Although other ideal dose settings or combination therapies using 5′-DFUR/capecitabine and cytostatics that exert TP up-regulation may exist, the problem remains to be seen in the optimal timing of administration of drugs in order to achieve maximum clinical effectiveness, since the process of TP up-regulation in tumour tissue by particular cytostatics takes a number of (4–6) days and TP levels gradually decrease after the discontinuation of treatment in the human cancer xenograft models (Sawada *et al*, 1998; Endo *et al*, 1999). There remain many other unresolved questions raised in this study. For example, why hormone refractory, receptor-positive breast cancers exhibited significantly higher sensitivity to the treatment, or why such a long median overall survival rate was obtained? These also need to be investigated. Further research is necessary.

In conclusion, the oral combination chemotherapy of 5′-DFUR and CPA exhibits low toxicity, and is a very convenient and effective treatment for metastatic breast cancer. This chemotherapy regimen may present a new treatment option for patients with metastatic breast cancer. Future clinical studies should compare this treatment with the existing standard treatment regimens for metastatic breast cancer.
